# An Innovative Approach to the Diagnosis of Cardiac Angiosarcoma

**DOI:** 10.7759/cureus.26323

**Published:** 2022-06-25

**Authors:** Samuel Kennedy, Michelle Dimza, Dennie Jones, Robert Seifert

**Affiliations:** 1 Department of Internal Medicine, University of Florida Shands Hospital, Gainesville, USA; 2 Department of Hematology and Oncology, University of Florida Shands Hospital, Gainesville, USA; 3 Department of Pathology, Immunology and Laboratory Medicine, University of Florida Shands Hospital, Gainesville, USA

**Keywords:** ctla4, pdl1, pd1, cardiac angiosarcoma, flow cytometry, immunohistochemistry

## Abstract

We present a case of a 32-year-old female presenting with shortness of breath and increasing oxygen requirements. Further imaging discovered a large mass extending circumferentially into the pericardium, cardiac wall, and chambers, involving the anterior and middle mediastinum. Direct tissue biopsy of the mass for a diagnosis was unsafe. Therefore, advanced flow cytometric analysis for tumor marker expression of the malignant effusion was used to differentiate the mass as a vascular sarcoma, consistent with cardiac angiosarcoma. Additionally, cytometric analysis for programmed cell death protein 1 (PD1), programmed death-ligand 1 (PDL1), and cytotoxic T-lymphocyte-associated antigen 4 (CTLA4) was also performed; a study seldom investigated for angiosarcomas but may have advantages over immunohistochemical analysis.

## Introduction

Angiosarcoma is a high-grade, aggressive tumor with a poor prognosis. Involvement of the mediastinum and heart is very rare and includes mostly isolated case reports. The differential diagnosis for malignant cardiac tumors includes primary sarcomas, metastatic tumors of the lung, breast, and kidney, as well as lymphomas and melanomas [1]. Various imaging modalities can be used to characterize the mass; however, a histopathologic examination is required for a definitive diagnosis. Unfortunately, due to the rarity and aggressive nature of cardiac angiosarcomas, there is no standard for therapy and proposed regimens remain controversial [2].

In this report, we present a case of a rapidly fatal mediastinal mass suspected to be a high-grade angiosarcoma. This case report highlights difficulties associated with early diagnosis and management strategies in such a rapidly progressive malignancy.

## Case presentation

A 32-year-old African American female with a past medical history of polycystic ovarian syndrome, uterine fibroids, and recent pericardial effusions, requiring pericardial windows, presented to the surgical intensive care unit as a hospital transfer to our facility for further workup of a complex mediastinal mass. The patient initially presented to an outside hospital two days prior complaining of one week of shortness of breath, fevers, and two months of weight loss. There, she was treated for pneumonia and had a chest tube placed for a large right pleural effusion. The patient had a history of two pericardial windows, six and one months prior to presentation. It was unclear what additional evaluation was performed at that time, and there was no indication of any prior positive cytology results from those effusions.

**Figure 1:**
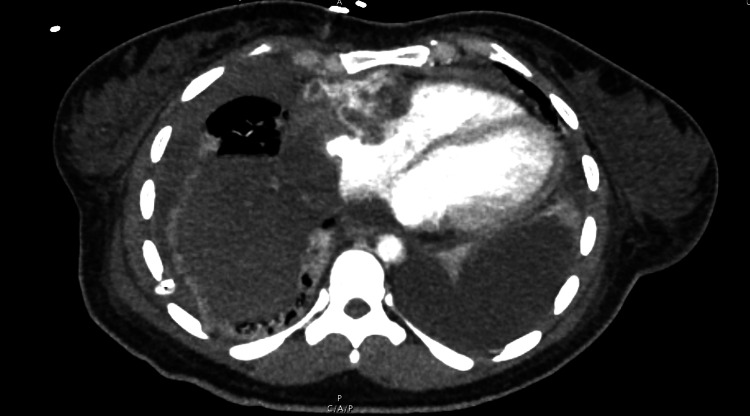
Chest CT showing large mass extending circumferentially into the pericardium, cardiac wall, and chambers

On admission, the patient was tachycardic at 150 beats per minute, febrile at 38.9°C, with a leukocytosis of 14,000 white blood cells per cubic millimeter with neutrophil predominance. The patient also had a new oxygen requirement of six liters per minute by nasal cannula. Computed tomography (CT) scanning showed a mediastinal tumor compressing the right ventricular wall and atrial wall and chamber (Figure 1). Transthoracic echocardiogram further defined the extension of the mass into the right atrium and ventricle. At this time, our differential diagnosis included Hodgkin's lymphoma, non-Hodgkin's lymphoma, and less likely, a germ cell tumor or small cell lung cancer. A thoracentesis specimen was submitted for flow cytometry and cytology with a gross description of clusters of neoplastic cells with high nuclear to cytoplasmic ratio, irregular nuclear membranes, and hyperchromasia.

**Figure 2:**
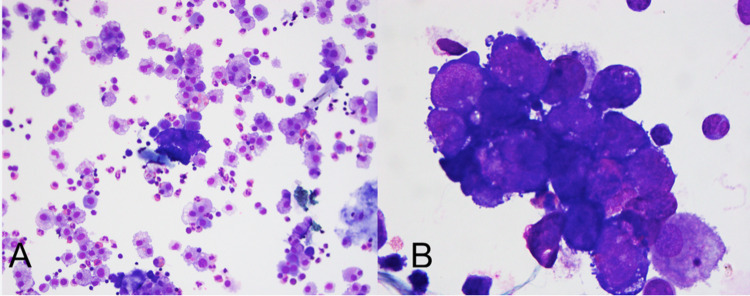
Wright-Giemsa-stained cytospin preparation of pleural fluid specimen at (A) 200x and (B) 1000x total magnification

**Figure 3:**
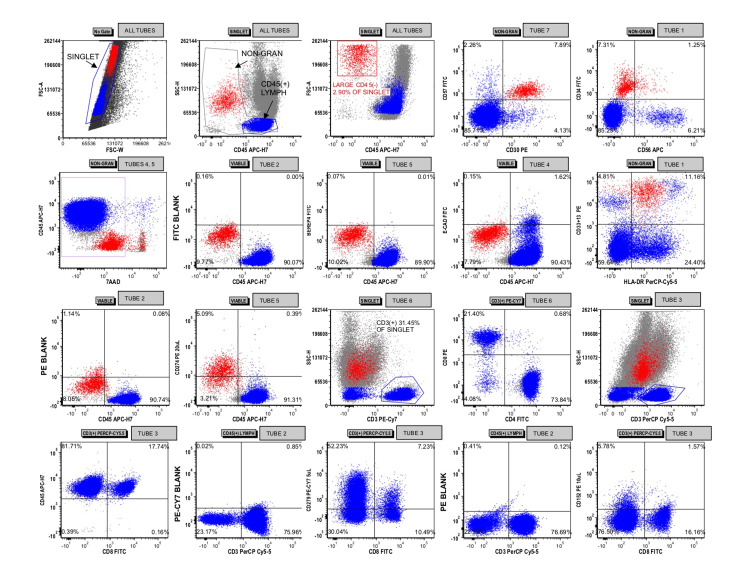
Flow cytometric analysis of the pleural fluid specimen Singlet events were gated with lymphocytes and non-granulocytes being identified across all tubes. A population of CD45(-) that was large in size by forward light scatter was gated and colored red. This population appeared to express CD30, CD57, CD33/CD13, and CD34. After first gating on viable events by 7-aminoactinomycin D (7-AAD) in tubes 4 and 5, these red-gated events were interrogated for CD274, BerEP4, and E-cadherin expression using the blank channels in tube 2 to demarcate negative/positive by the quadrants shown. CD3(+) T cells were gated with relative quantification of CD8(+) and CD4(+) subsets shown via tube 6. A similar proportion of T cell subsets was obtained in tube 3, which did not include CD4 antibodies. Expression of CD279 and CD152 were interrogated on the T cells, using the blank channels in tube 2 to demarcate negative/positive by the quadrants shown.

Cytology identified malignant cells (Figure 2) immunoreactive for ERG and CD31 with negative immunohistochemistry for cytokeratin, desmin, S-100, ALK1, and CD30. Flow cytometric analysis detected a population of CD45(-) cells that lacked expression of BerEP4 and E-cadherin (Figure 3). They were positive for CD34, CD30, CD33, CD13, and CD57 but negative for CD56 and dim positive for HLA-DR. CD274 (programmed death-ligand 1 (PDL1)) appeared dimly positive in the malignant cell population. Together, these findings were concerning for a vascular neoplasm, consistent with angiosarcoma.

Flow cytometric analysis for programmed cell death protein 1 (PD1), PDL1, and cytotoxic T-lymphocyte-associated antigen 4 (CTLA4) was also performed on this specimen (Table 1). The malignant cells were dimly positive for PDL1 with only a small subpopulation of CD8(+) T cells (1.27% of total events) being PD1(+). The CD4:CD8 ratio of T cells in the specimen was approximately 4.7:1. CTLA4 was essentially negative in the T cells.

**Table 1:** Antibody panel for evaluation of PD1, PDL1, and CTLA4 PD1: programmed cell death protein 1; PDL1: programmed death-ligand 1; CTLA4: cytotoxic T-lymphocyte-associated antigen 4; BD: Becton Dickinson; (Blank): no antibody of this fluorophore; 7AAD: 7-aminoactinomycin D used for viability gating; FITC: fluorescein isothiocyanate; PE: phycoerythrin; PerCP-Cy5.5: peridinin chlorophyll protein-cyanine 5.5; APC: allophycocyanin; APC-H7: allophycocyanin-cyanine tandem conjugate; PE-Cy7: phycoerythrin-cyanine 7.

Fluorochrome		FITC	PE	PerCP-Cy5.5	APC	APC-H7	PE-Cy7
Tube 1	Label	CD34	CD13 + CD33	HLA-DR	CD56	CD45	CD14
	Clone	8G12	L138/P67.6	L243	NCAM16.2	2D1	RMO52
	Amount	5 uL	5/5 uL	10 uL	5 uL	3 uL	5 uL
	Vendor	BD	BD/BD	BD	BD	BD	Beckman Coulter
Tube 2	Label	(Blank)	(Blank)	CD3	(Blank)	CD45	(Blank)
	Clone			SK7		2D1	
	Amount			10 uL		3 uL	
	Vendor			BD		BD	
Tube 3	Label	CD8	CD152	CD3	CD28	CD45	CD279
	Clone	SK1	BNI3	SK7	CD28.2	2D1	EH12.1
	Amount	5 uL	5 uL	10 uL	5uL	3 uL	5 uL
	Vendor	BD	BD	BD	Beckman Coulter	BD	BD
Tube 4	Label	E-CAD	CD274	7AAD	(Blank)	CD45	(Blank)
	Clone	67A4	MIH1	N/A		2D1	
	Amount	10 uL	20 uL	5 uL		3 uL	
	Vendor	Life Technologies	BD	BD		BD	
Tube 5	Label	BEREP4	CD274	7AAD	(Blank)	CD45	(Blank)
	Clone	Ber-EP4	MIH1	N/A		2D1	
	Amount	10 uL	20 uL	5 uL		3 uL	
	Vendor	DAKO	BD	BD		BD	
Tube 6	Label	CD4	CD8	CD5	CD7	CD45	CD3
	Clone	SK3	SK1	L17F12	M-T701	2D1	UCHT1
	Amount	5 uL	5 uL	10 uL	5 uL	3 uL	2.5 uL
	Vendor	BD	BD	BD	BD	BD	Beckman Coulter
Tube 7	Label	CD57	CD30	CD4	CD25	CD45	CD3
	Clone	HNK-1	HRS4	SK3	2A3	2D1	UCHT1
	Amount	5 uL	5 uL	5 uL	5 uL	3 uL	2.5 uL
	Vendor	BD	Beckman Coulter	BD	BD	BD	Beckman Coulter

Despite the patient’s young age and lack of additional comorbidities, it was felt that they were not a candidate for an attempted surgical resection, especially with positive pleural fluid cytology, indicating tumor dissemination. Oncology was consulted and upon discussion of risks and benefits, the patient decided to attempt treatment with palliative chemotherapy. Though the patient was treated with antibiotics and diuretics, she worsened clinically on the fifth day with new fevers, and increased oxygen requirement with questionable pneumonia, as well as pulmonary edema, which delayed the initiation of chemotherapy. For the next few days, the patient continued to decompensate further with worsening hypoxia and intermittent hemoptysis and was transferred to the medical intensive care unit. There were extensive goals of care discussions between the patient, their family, oncology, internal medicine, and palliative care. On hospital day eight, the patient chose to pursue comfort care. The patient passed away on hospital day nine and the family declined a post-mortem exam.

## Discussion

Mediastinal and cardiac involvement by malignant vascular neoplasms is uncommon. Furthermore, the extent of invasion to involve the middle and anterior mediastinum, pericardium, and both right-sided cardiac chambers as seen in this case is exceedingly rare. Only 25% of primary cardiac tumors are malignant, and of those, soft tissue sarcomas are the most common [1]. Still, the incidence of a primary cardiac sarcoma is exceedingly rare [3].

Cardiac sarcomas have been found predominantly in males between the ages of 30-50 years old. They most commonly involve the right side of the heart. Therefore, patients most often present with signs and symptoms of right-sided heart failure. Pericardial effusion, dyspnea, chest pain, palpitations, and myalgia are common presenting complaints. Angiosarcoma can also be a rare etiology for cardiac tamponade [4,5].

Our patient required two pericardial windows prior to the presentation, with unknown fluid analyses, suggesting a relatively indolent early clinical course, in contrast to a rapid decline after diagnosis. During hospitalization, the patient experienced intermittent hemoptysis and had ground-glass opacities on CT imaging of the lungs (Figure 1). Although these are nonspecific findings, reports of patients presenting with diffuse alveolar hemorrhage secondary to mediastinal angiosarcomas have been cited and can represent rapid progression [6,7].

Chest CT with intravenous contrast is often the first and possibly the only imaging modality required for evaluation. Chest and cardiac gated magnetic resonance imaging, positron emission tomography, or echocardiography may provide benefits for diagnostic or treatment purposes. Ultimately, a tissue sample is required for definitive diagnosis [1]. Unfortunately, due to the location and invasive nature of our patient’s mass, we were not able to get a direct tissue sample.

Therefore, cytologic and flow cytometric evaluation of the malignant effusion was key to diagnosing this patient as the expression of ERG, CD31, and CD34 with negative melanoma, lymphoma, and carcinoma markers is consistent with vascular differentiation, not myeloid blast differentiation.

Currently, PD1 and PDL1 evaluation in certain tumor specimens is performed by specialized immunohistochemical techniques looking for PDL1 immunoreactivity in tumor cells [8]. Our flow cytometry lab evaluates PD1, PDL1, and CTLA4 expression when a non-hematopoietic malignancy is considered. In this specimen, the malignant cells dimly expressed PDL1 and there were relatively few CD8(+) and PD1(+) T cells. Unfortunately, no immunohistochemical analysis for PDL1 was performed for correlation. PD1 checkpoint inhibition has seldom been investigated in angiosarcoma and may have therapeutic utility [9,10]. There is no current consensus on the effectiveness of such treatment and the impact of PDL1 expression on prognosis is controversial [9,11-13]. It is not possible to draw conclusions about prognosis or potential therapies in this case, but this finding remains interesting and not well studied in the literature. Flow cytometry, unlike immunohistochemistry, was able to show that the majority of the PD1(+) T cells were CD4(+) with only a small subset of CD8 T cells being PD1(+). Immunohistochemical assays for PD1 in tumor-infiltrating lymphocytes do not typically separate CD4(+) from CD8(+) tumor-infiltrating T cells and CD8 T cells are thought to be the chief effector in tumor-related immunosuppression [8]. Thus, an immunohistochemical assay could, in our specimen, incorrectly predict the presence of effective PD1(+) tumor-infiltrating lymphocytes to be the target of the blockade.

Unfortunately, the prognosis for patients with malignant vascular tumors is dismal. Treatment strategies vary but none have produced substantial long-term success. Surgical resection has shown the most benefit in survival. One study found that for those patients who are candidates for complete resection, there is a median overall survival of 12 months, compared to those with incomplete resection or no resection, who experience a median survival of one month [2]. Due to the propensity for sarcomas to metastasize early, adjuvant chemotherapy is often recommended, though it has yet to demonstrate conclusive benefit. Combinations of cyclophosphamide, vincristine, dacarbazine, ifosfamide, methotrexate, and doxorubicin have been used with only isolated reports of sustained response [14]. Of these, doxorubicin with ifosfamide is the most commonly offered regimen for soft tissue sarcomas. However, the underlying specific histology of the sarcoma is important. Although radiation has shown success for mediastinal sarcomas, the myocardium is very sensitive to radiation and far too risky in the setting of a primary cardiac sarcoma [15]. Lastly, heart transplantation has been explored for these patients but is typically not a viable option due to aggressive and early metastasis [16].

## Conclusions

This case underscores the many challenges surrounding the diagnosis and management of mediastinal sarcomas. A high level of diagnostic suspicion for young patients with cardiac complaints is the most important link in the evaluation. Complete surgical resection with a multimodality oncologic treatment is appropriate for an aggressive approach. At present, systemic therapy still has limited benefits, but may be useful in providing some palliation.

## References

[1] Paraskevaidis IA, Michalakeas CA, Papadopoulos CH, Anastasiou-Nana M (2011). Cardiac tumors. ISRN Oncol.

[2] Hamidi M, Moody JS, Weigel TL, Kozak KR (2010). Primary cardiac sarcoma. Ann Thorac Surg.

[3] Reynen K (1996). Frequency of primary tumors of the heart. Am J Cardiol.

[4] Habibi R, Faramarzi N, Altamirano AJ, Dadkhah S (2018). A patient presenting with cardiac tamponade and the challenges of finding its cause: a cardiac angiosarcoma. Case Rep Cardiol.

[5] Pacha HM, Soud M, Alraies MC (2018). Beyond Beck's triad: a rare cause of cardiac tamponade and hemoptysis. Ochsner J.

[6] Datta D, Gerardi DA, Lahiri B (2018). Mediastinal angiosarcoma presenting as diffuse alveolar hemorrhage. Respir Med Case Rep.

[7] Wang HP, Shi JH, Wang WZ, Zhang L (2018). Pulmonary metastatic angiosarcoma presenting with diffuse alveolar hemorrhage: 9 case reports. (Article in Chinese). Zhonghua Nei Ke Za Zhi.

[8] Conroy JM, Pabla S, Nesline MK (2019). Next generation sequencing of PD-L1 for predicting response to immune checkpoint inhibitors. J Immunother Cancer.

[9] Boichard A, Wagner MJ, Kurzrock R (2020). Angiosarcoma heterogeneity and potential therapeutic vulnerability to immune checkpoint blockade: insights from genomic sequencing. Genome Med.

[10] Sindhu S, Gimber LH, Cranmer L, McBride A, Kraft AS (2017). Angiosarcoma treated successfully with anti-PD-1 therapy - a case report. J Immunother Cancer.

[11] Bagaria SP, Gatalica Z, Maney T (2018). Association between programmed death-ligand 1 expression and the vascular endothelial growth factor pathway in angiosarcoma. Front Oncol.

[12] Botti G, Scognamiglio G, Marra L (2017). Programmed death ligand 1 (PD-L1) expression in primary angiosarcoma. J Cancer.

[13] Kösemehmetoğlu K, Özoğul E, Babaoğlu B, Tezel GG, Gedikoğlu G (2017). Programmed death ligand 1 (PD-L1) expression in malignant mesenchymal tumors. Turk Patoloji Derg.

[14] Llombart-Cussac A, Pivot X, Contesso G (1998). Adjuvant chemotherapy for primary cardiac sarcomas: the IGR experience. Br J Cancer.

[15] Reardon MJ, Walkes JC, Benjamin R (2006). Therapy insight: malignant primary cardiac tumors. Nat Clin Pract Cardiovasc Med.

[16] Li H, Yang S, Chen H, Yang Z, Hong T, Hou Y, Wang C (2016). Survival after heart transplantation for non-metastatic primary cardiac sarcoma. J Cardiothorac Surg.

